# Perceptual Connectivity Influences Toddlers’ Attention to Known Objects and Subsequent Label Processing

**DOI:** 10.3390/brainsci11020163

**Published:** 2021-01-27

**Authors:** Ryan E. Peters, Justin B. Kueser, Arielle Borovsky

**Affiliations:** 1 Department of Psychology, The University of Texas at Austin, Austin, TX 78712, USA; 2 Department of Speech, Language & Hearing Sciences, Purdue University, West Lafayette, IN 47907, USA; jkueser@purdue.edu (J.B.K.); aborovsky@purdue.edu (A.B.)

**Keywords:** perceptual knowledge, shared features, language development, lexicosemantic development, semantic networks, visual object processing, word processing, attentional biases

## Abstract

While recent research suggests that toddlers tend to learn word meanings with many “perceptual” features that are accessible to the toddler’s sensory perception, it is not clear whether and how building a lexicon with perceptual connectivity supports attention to and recognition of word meanings. We explore this question in 24–30-month-olds (*N* = 60) in relation to other individual differences, including age, vocabulary size, and tendencies to maintain focused attention. Participants’ looking to item pairs with high vs. low perceptual connectivity—defined as the number of words in a child’s lexicon sharing perceptual features with the item—was measured before and after target item labeling. Results revealed pre-labeling attention to known items is biased to both high- and low-connectivity items: first to high, and second, but more robustly, to low-connectivity items. Subsequent object–label processing was also facilitated for high-connectivity items, particularly for children with temperamental tendencies to maintain focused attention. This work provides the first empirical evidence that patterns of shared perceptual features within children’s known vocabularies influence both visual and lexical processing, highlighting the potential for a newfound set of developmental dependencies based on the perceptual/sensory structure of early vocabularies.

## 1. Introduction

Does a toddler’s knowledge of and attention to the subcomponents of word meanings influence lexicosemantic processing? Research on adults suggests the answer is “yes”. Findings show different aspects of word meanings influence a range of adult psycholinguistic processes, including categorization [1,2], word/concept learning [3], and semantic priming [4,5]. Meanwhile, recent efforts to explore the effects of subcomponents of word meanings on toddlers’ normative vocabulary development have revealed not all types of meaning are equally important: perceptual features matter most [6,7]. However, less is known about whether and how perceptual aspects of word meanings influence lexical processing in early language development.

A learner’s knowledge of a word (e.g., bus) can be described using semantic features (e.g., is yellow, used by schools, a vehicle), which can be derived by collecting semantic feature production norms [8]. Such empirically derived features have contributed to our contemporary understanding of adult lexicosemantic processing [1,2,3,4,5,9,10,11,12,13] and have more recently also been applied to the study of lexical development in toddlerhood [6,7,14,15,16,17,18,19]. Semantic features can be categorized into several possible types [20], of which perceptual, functional, and taxonomic features have been of particular interest to developmental researchers.

Perceptual features about a word’s meaning are defined as information accessed via a primary sensory channel (e.g., is yellow, has wheels, is large). Numerous theoretical accounts suggest early lexicosemantic processing is driven by the perceptual features of objects, while more abstract features and relations only influence processing after substantial representational enrichment [21,22,23,24,25,26,27,28,29]. For example, when learning the meaning of the word “bus”, these accounts argue a child first identifies perceptual features, and then later, with the aid of language [23], adds more abstract features such as its functions (e.g., used for passengers, used by schools) and taxonomic relations with other words (e.g., a vehicle).

The early importance of perceptual experience has been highlighted in work on conceptual processing [30,31,32,33,34,35] and word learning [36,37,38,39,40]. A common approach contrasts the influence of object form and function on tasks such as object exploration [30] or novel name generalization [37] across development. Object form has pervasive impacts across development and task type, but object function effects are limited to older children and adults. A second common approach compares how children recognize featural regularities in unfamiliar objects when forming categories. In these studies, even very young children recognize shared perceptual features among objects [31,34,41], but have difficulties recognizing and using more abstract conceptual features [30,32,33,34].

While this work shows young children recognize and use shared perceptual features among observed objects, it is less clear whether they likewise make use of features shared with words in their own lexicon. Evidence relevant to this overarching question that motivates the current study comes from a growing body of work employing graph-theoretic noun–feature network modeling approaches to the study of lexicosemantic development [6,7,16]. Models of early linkages among word meanings in early lexical development have revealed that characterizing semantic connections between words by perceptual features (as opposed to functional or taxonomic features) results in a lexical network with dense links [7,17] that contain clusters that resemble adult superordinate taxonomic designations (e.g., animals, clothes, food, etc.; [17]), and most strongly relate to the age of acquisition (AoA) of early learned words [6,7]. Moreover, words that have more perceptual semantic features (i.e., higher perceptual degree) are learned earlier, even when accounting for word frequency in child-directed speech [7]. This consistent contribution of perceptual lexicosemantic structure on patterns of normative development further supports the hypothesis that shared perceptual features within the minds of individual children influence lexical processing.

However, evidence from relevant empirical work is less straightforward. Findings from a priming study comparing the processing time course of words following primes sharing different aspects of word meanings suggest that some perceptual information (like color) is not prioritized over semantic (taxonomic) information in real-time processing [42]. However, a second study suggests that having some perceptual connectivity (organized by shape) may influence how toddlers attend to some perceptual features like color [43]. Further indirect evidence comes from a study exploring the effects of category density on the processing of familiar words [44]. Using a variant of the Looking While Listening Paradigm (LWLP; [45])—which indexes online processing via measurements of eye gaze to visual images of objects accompanied by their spoken labels—Borovsky and colleagues found that 2-year-old toddlers show facilitated processing for words that are members of categories that they are relatively more knowledgeable of. Neural network simulations [46] provide a potential explanation for why dense patterns of shared features might be important for the processing of known words. Namely, lexicosemantic systems with more interconnected semantic structure are more easily able to recognize and map similarities (i.e., shared features), resulting in richer, more robust representations and more fluid processing.

Nevertheless, this account leaves one thing unclear: the timing of the facilitatory effects. While these effects may take place during lexical processing due to more robust representations, another possibility is that greater connectivity may also serve to draw attention to an object before lexical processing starts. Such biased attention to an object pre-labeling could in turn facilitate subsequent label processing. Borovsky and colleagues [44] used counterbalanced stimuli to account for any potential effects of pre-labeling attentional biases. However, while such counterbalancing can account for random biases, it cannot account for biases that regularly occur in the same direction as the effect of interest.

The hypothesis that perceptual density influences pre-labeling attentional biases is particularly plausible given empirical evidence linking the speed of visual object processing and sensitivity to holistic shape to word knowledge [47,48] and experience with same category items [49]. Specifically, infants show a developmental shift from slower, piecemeal processing of local perceptual features to faster, more holistic processing as they gain relevant experience and knowledge. While explanations for this shift often frame it in terms of increasing abstraction, e.g., [50], another non-exclusive explanation parallels the one above [46]. Namely, greater knowledge of objects with perceptual similarities (e.g., same category items) allows for easier recognition and mapping of shared perceptual features, resulting in richer, more coherent representations and faster, more holistic processing. In other words, for items with greater perceptual connectivity, we might see faster, more holistic processing that biases attention to such objects relative to items with lower perceptual connectivity and slower, more piecemeal processing. Thus, in the current study, we aim to explore not only the effects of a word’s perceptual connectivity on lexical processing, but also the effects of connectivity-driven attentional biases pre-labeling.

### 1.1. Modeling Lexicosemantic Development Using Noun–Feature Networks

**Figure 1:**
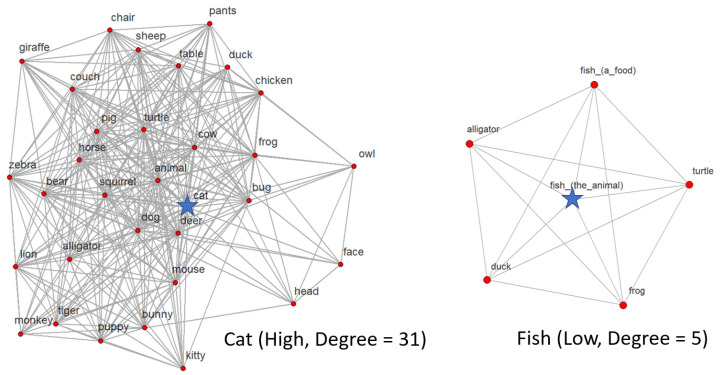
The neighborhoods for “cat” and “fish” (marked by blue stars), extracted from a normative 26-month-old’s (visual–motion and visual–form and surface) perceptual network.

Noun–feature networks can be visualized as a set of nodes and links (see Figure 1). The nodes represent noun-concepts, and the links represent shared features according to a set of feature production norms (e.g., [8]. Features of different types (e.g., perceptual vs. functional) can be used to decompose semantic networks into separate feature-specific networks to explore how different types of features drive early lexicosemantic development. Graph-theoretic methods can then be applied to these networks to characterize structural patterns. For example, the most foundational graph-theoretic metric, degree, characterizes the connectivity of a node based on the number of links it has with other nodes. For lexicosemantic networks, degree is equivalent to semantic neighborhood density, a metric that explains variance in empirical findings in studies of semantic richness [10], word learning [51,52], and word processing [53].

### 1.2. Participant Differences That May Relate to Perceptual Connectivity Effects

Altogether, prior evidence supports the hypothesis that a word’s semantic connectivity, particularly with respect to perceptual features, may facilitate its recognition in children. However, this effect may not be the same for all children. A child’s specific knowledge, life experiences, and temperament influence their language learning and processing [54,55]. As such, we consider three potentially relevant individual differences: age, word learning skill, and temperamental tendency to maintain focused attention.

The age of a child might influence or mediate perceptual connectivity and lexical processing for two reasons. First, there is growing evidence that children undergo a developmental shift in lexicosemantic structure as they approach two years of age. While 2-year-olds show reliable semantic priming [56,57], only slightly younger children (18–21 months) do not [58,59,60]. If this shift is driven by maturational differences, we might likewise expect age to relate to differences in the recognition and use of shared perceptual features. Second, age constrains children’s history of language learning experiences in a variety of ways. For example, older children are both more likely to have been exposed to a wider variety of experiences and more likely to have had access to a greater depth of information in those experiences due to more developed memory and attentional systems, e.g., [24]. Thus, older children may be more likely to recognize shared features and reap the facilitatory effects on processing.

A child’s skill in learning words might relate to perceptual connectivity effects for similar reasons to age. Indeed, the aforementioned developmental shift in semantic priming appears to be driven in part by word knowledge—with younger 18- to 20-month-olds with larger vocabularies showing semantic priming similar to 2-year-olds, while those with smaller vocabularies do not [59,60]. Likewise, children who are better word learners and have more knowledge than their lower-skill peers will likely have better processing of the semantic content in language. Better word learners, then, would be expected to reap more benefits from semantic connectivity than poorer word learners. While word knowledge varies with age, measuring word learning skill in comparison to same-age peers—via vocabulary size percentile—allows us to explore orthogonal variance in effects.

Finally, individual differences in focused attention may lead to differences in depth of processing and learning opportunities. Attention has been described as a gatekeeper that determines what information in the environment becomes input to learning mechanisms [61]. However, all attentional skills may not be equally valuable for learning. Sustained visual attention to label targets is particularly beneficial for learning object–label mappings (as compared to joint attentional skills; [62,63,64,65]). While much of this work is based on measures extracted from looking patterns, to separate our variables of interest, here we use a parent-report measure of temperamental tendency to maintain focused attention (hereafter referred to as focused attention skill), which has shown robust relations to individual differences in word learning outcomes [66,67]. This measure is one subscale from the Early Childhood Behavior Questionnaire (ECBQ; [68]), a validated measurement of fine-grained aspects of toddler temperament. Questions on the scale aim to measure how focused children generally are during everyday activities such as toy play and book reading—routine activities that provide regular opportunities for language learning and processing. Thus, age, word learning skill, and tendency to maintain focused attention may all relate to individual differences in children’s language learning histories—with cascading consequences for the make-up of their lexicosemantic systems and corresponding variation in their attention to and recognition of familiar objects and words. 

### 1.3. The Current Study

The primary goal of this project is to explore how the perceptual connectivity of familiar objects influences patterns of pre-labeling attentional biases and subsequent word processing. A secondary goal is to determine whether any perceptual connectivity effects are related to differences in age, word learning skill, or focused attention skill. To test these pre-registered questions, we exposed a large sample (*N* = 60) of 24- to 30-month-old toddlers to pairs of objects whose labels (e.g., airplane) these children had in their productive vocabulary. These object–label pairs varied in their perceptual connectivity to other words as measured using a set of feature production norms for early learned nouns [69]. The objects in each pair came from the same semantic category (i.e., animals, vehicles) so that each object differed from the other only in connectivity of perceptual features and no other feature types (e.g., taxonomic or functional). We measured pre- and post-labeling attention to the high- and low-connectivity object in each pair using eye tracking. 

We hypothesized higher perceptual connectivity would increase pre- and post-labeling fixations to objects. Furthermore, we reasoned that the relation between pre- and post-label looking would provide informative theoretical insights about the mechanisms that drive lexical acquisition in relation to perceptual connectivity: If perceptual connectivity largely drives non-linguistic attentional biases that lead to learning, then we would expect that the relation between perceptual connectivity on post-label looking would disappear after controlling for pre-label looking. Conversely, if perceptual connectivity supports attentional biases and lexical development individually, then we would expect that a significant effect of perceptual connectivity on post-labeling looking patterns would remain even after accounting for pre-labeling attentional biases.

## 2. Materials and Methods

### 2.1. Participants

Sixty 24- to 30-month-old toddlers were recruited for this study. We chose this age range because it is a time of particularly dramatic change in children’s lexicosemantic systems. We did not include younger toddlers because as productive vocabulary sizes become smaller so too do differences in patterns of connectivity—making it difficult to choose balanced stimuli. Six children completed the study but were not included in the analyses for the following reasons: history of chronic ear infections and hearing loss concerns (*N* = 2), neurological impairment (*N* = 1), and insufficient data due to track-loss/fussiness (*N* = 3). Fifty-four toddlers were retained in the final analyses (26 female, 28 male), all of whom met the following pre-registered (see Appendix A) inclusionary criteria: normal vision and hearing; no history of chronic ear infections; no history of neurological or cognitive impairments; not born preterm (<37 weeks) or with low birth weight (<5 lb 8 oz); not exposed to language other than English for more than 8 h a week (~10% of waking hours).

### 2.2. Semantic Connectivity Measure

#### 2.2.1. Features

We drew semantic features from feature production norm datasets [8,69] that together, include all 359 nouns concepts on the MacArthur Bates Communicative Developmental Inventory, Words and Sentences from (MBCDI:WS, [70]). The features are classified into four broad types (encyclopedic, functional, perceptual, and taxonomic) according to the Cree and McRae [20] Brain region knowledge type taxonomy. Perceptual features can be further broken up into an additional seven subtypes: smell, sound, tactile, taste, visual–color, visual–form and surface, and visual–motion. Only two types of perceptual features—visual–form and surface and visual–motion features—are used in this work based on modeling evidence that they are the primary types of perceptual features that predict acquisition patterns in early word learning [7].

#### 2.2.2. Network Construction

We constructed graph-theoretic lexicosemantic networks for each participant, based on the nouns they were reported to say on the MBCDI:WS. While productive vocabulary accounts for only a subset of word knowledge, it has consistently related with individual differences in lexical and lexicosemantic processing, e.g., [44,59]. Thus, following other recent work employing the noun–feature network methodology [6,7,14,15,16,17,18,19], nodes in each child’s network represented known nouns. Links between nodes represented at least one shared perceptual feature, and were treated as unweighted, undirected edges, following precedent [6,7,16].

We also created a normative 26-month-old’s network containing all nouns with an Age of Acquisition (AoA) of less than or equal to 26 months. We calculated AoA for each of the 359 concrete nouns on the MBCDI:WS using the Wordbank database [71]—an open repository of MBCDI data, of which we used 5450 administrations of the MBCDI:WS. Following Braginksy, Yurovsky, Marchman, and Frank [72], for each word, we calculated the proportion of children who produced the word at each age (ranging from 16 to 30 months). We then fit logistic curves to the proportion data for each word and took the age at which the curves crossed. 5 as a word’s AoA.

#### 2.2.3. Calculating Semantic Connectivity

This project focuses on one of the simplest network metrics: degree. The degree of a word is simply defined as the number of links it has to other words in the network. Given that links are defined using shared features, degree characterizes the pattern of shared features between a word and its neighbors. We calculated the normative degree of each item as its degree in the normative 26-month-old’s perceptual network described above. As described in detail in the next section, paired items were rank ordered in terms of normative degree and assigned to either “high” or “low” normative degree categories. Figure 1 shows one such item pair and their respective neighborhoods (i.e., the full set of words in the network they share a link with).

We also calculated item degree based on each individual child’s vocabulary. However, preliminary analyses revealed that for all but a single item pair for a single participant, the rank ordering of normative degree items aligned with the rank ordering of individually calculated degree. Therefore, to simplify our results and inferences, we limit our focus to normative degree.

### 2.3. Materials

#### 2.3.1. Item Selection

Words were chosen from the animal and vehicle categories in the MBCDI: Words and Sentences. These words were presented as part of a larger study that included novel objects, which are not analyzed here. We used the Wordbank database to select six highly known words in each category (i.e., produced by at least 75% of 26-month-olds) for a total of 12 words. The items were then organized into yoked pairs, with three pairs of animals (bug–chicken, bear–duck, catfish) and three pairs of vehicles (bus–boat, truck–bike, airplane–train). Pairs were selected so that one item had a high perceptual degree, calculated using the normative 26-month-old’s network, and one had a low perceptual degree (see Figure 1).

Furthermore, the yoked pairs were chosen to control for a range of other factors that have potential influence on patterns of psycholinguistic processing. The factors that we controlled for included total degree (i.e., degree in a network with connections determined using features of all types), total number of perceptual features, total number of visual–form and surface and visual–motion features, word frequency in child-directed speech in the North American English language corpora of CHILDES with target children no older than 30 months [73], number of phonemes, number of syllables, bigram frequency, phonological neighborhood density, AoA, and proportion of children producing the word at 26 months of age. T-tests also confirmed that all controlled factors were not significantly different across pairs (see Appendix A for details).

#### 2.3.2. Auditory Stimuli

A female Standard American English speaker spoke in an infant-directed voice for all auditory experimental stimuli and additional encouraging phrases. The spoken stimuli were recorded on a mono channel at 44.1 kHz sampling rate. All experimental stimuli were adjusted to a mean duration of 1016 ms, and all stimuli, including encouraging phrases and an attention getter, were standardized to a mean intensity of 70 dB.

#### 2.3.3. Visual Stimuli

**Figure 2:**
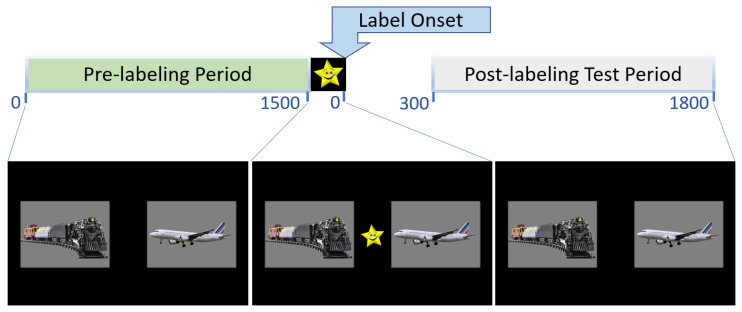
Example of visual stimuli in an experimental trial. Each trial began with a pre-labeling period, during which target and distractor images appeared alone, side by side. After 1500 ms, a salient central image (e.g., smiling star) appeared. Simultaneously, the attention getting auditory stimulus “Look!” was presented. Once the toddler fixated on the central image for 100 ms, it disappeared. Then, the spoken label for the target was presented, followed by an encouraging phrase (e.g., “Train! You’re doing great!”). The post-labeling test period then went from 300 to 1800 ms post naming onset.

Visual experimental stimuli consisted of 600 × 450-pixel photorealistic color images on a 1920 × 1080-pixel screen. Object images were selected so as to match toddlers’ experiences with the experimental items, verified by consulting with parents and laboratory members. All images were isolated on a grey background. Figure 2 shows an example of the visual stimuli in an experimental trial. Other images were also used to keep and direct the attention of the children throughout the study. These images included large pictures of characters from popular children television shows and small images used to direct the children’s attention to the center of the screen.

#### 2.3.4. Eye-Tracking Procedure and Apparatus

For the eye-tracking task, the child was seated in a toddler car seat in front of a 24-inch monitor. The parent sat to the left and slightly behind the child, while an experimenter sat to the right so as to be able to encourage the child to attend to the screen. Before the experiment, parents were asked to refrain from naming or describing any of the images on the screen.

The tracker was calibrated using a five-point procedure with a looming bulls’ eye image paired with a whistling sound. For the experimental task, each trial began with a small (30 × 30-pixel), colorful gaze-contingent central image presented on a black background. The image disappeared after the child fixated on it, and was replaced by the target and distractor images, which were displayed side by side (see Figure 2). After 2000 ms, another colorful central image appeared (100 × 100-pixel). Simultaneously, the attention getting auditory stimulus “Look!” was presented. Once the toddler fixated on the central image for 100 ms, it disappeared. Then, the spoken label for the target was presented, followed by an encouraging phrase (e.g., “Train! You’re doing great!”). The target and distractor images were displayed on the screen for 4000 ms post-label onset.

Each experimental item appeared four times across the experiment, counterbalanced so that it appeared twice as the target and twice as the distractor, once on each side of the screen for both. There were 24 experimental trials, distributed across three blocks of eight trials. In each block, there were also four trials containing novel objects. These trials were part of a separate study and are not discussed further in this paper. Additionally, every four trials, children saw filler trials that contained scenes with popular characters. These scenes were accompanied by encouraging phrases (e.g., “Wow! Look at that!”). The entire eye-tracking procedure lasted approximately 15–20 min.

The movements of participants’ right eyes were recorded from image onset to offset at 500 Hz, using an SR-Research Eyelink 1000 plus eye tracker. Movements were binned into 50 ms intervals and classified as fixations, saccades, and blinks following the default setting on the eye-tracker. Finally, target and distractor areas of interest were defined as the two 650 × 400-pixel areas, where the corresponding images were displayed.

#### 2.3.5. Offline Assessments

Before coming to the lab, parents were asked to complete an online version of the MBCDI:WS. After the eye-tracking task, parents completed three questionnaires—a background history form, a vocabulary checklist for the experimental items, and the short form of the Early Childhood Behavior Questionnaire (ECBQ; [68]).

For the background history questionnaire, parents were asked to provide demographic information and answered questions regarding their toddler’s language environment. For the vocabulary checklist, parents rated their child’s comprehension and production for all of the experimental items on a scale of 1 (“child definitely does not understand/say the word”) to 4 (“child definitely understands/says the word”). Items for which parents rated comprehension as less than “3” were removed from subsequent analyses. Finally, the ECBQ measured a range of temperamental characteristics of the child by presenting a description of a behavior and asking the parent to describe how often they had observed their child doing the behavior in the last two weeks on a scale from 1 (never) to 7 (always). For example, one question asked, “When engaged in play with his/her favorite toy, how often did your child play for more than 10 min?” We limited our analyses to only include the attentional focusing subscale.

**Figure 3:**
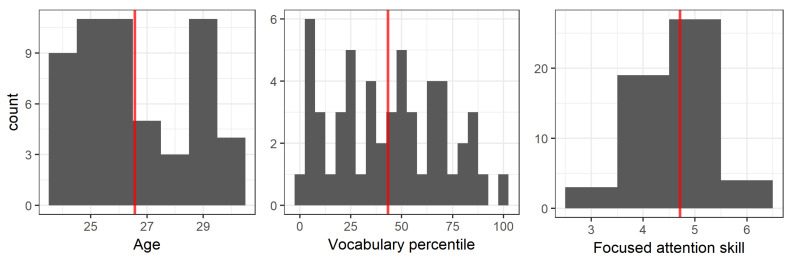
Histograms for age, vocabulary percentile and focused attention skill. Red lines show means.

These offline assessments were then used to calculate three participant measures: age, productive vocabulary percentile, and focused attention skill. The histograms, descriptive statistics, and pairwise correlations for these three measures are presented in Figure 3, Table 1A,B, respectively. Notably, the significant correlation between vocabulary percentile and focused attention skill, *r* = 0.35, *t*(51) = 2.6, *p* = 0.01, replicates previous findings [66,67].

#### 2.3.6. Data Cleaning

As laid out in our pre-registration (see https://osf.io/ha48g/Pre-registration: https://osf.io/ha48g/?view_only=29e499d157114f87afb7633dd06f34d0), individual trials were removed if the percentage of track-loss exceeded 80 percent, following prior precedent (e.g., [44]). This resulted in the removal of 49 trials (3.8%) from pre-labeling period analyses and 18 trials (1.4%) from post-labeling test period analyses. Trials were also removed from analyses if parents rated comprehension for either item as less than “3” on the targeted vocabulary checklist. This resulted in the removal of 77 trials (6.3%). Additionally, the data for individual participants were removed if, after the removal of trials based on track-loss and the vocabulary checklist, they did not have at least two high-connectivity and two low-connectivity data points (*N* = 3).

## 3. Results

### 3.1. Approach to Analyses

We conducted three sets of parallel analyses on two predefined time windows of 1500 ms in duration: (1) the pre-labeling period (image onset to 1500 ms) and then, (2) on the pre-registered post-labeling test period (300 to 1800 ms post-label onset)—corresponding with time windows used in other studies of infant known-word recognition (e.g., [44,45]). This time window departs from those laid out in our pre-registration. Our pre-registered post-labeling time windows were chosen based on novel time course effects in our pilot sample (*N* = 6). However, the time courses in our final sample more closely resembled those typically seen in Looking While Listening experiments of familiar word processing for children of this age. Thus, to accommodate this mistaken decision based on a small sample while reducing our degrees of freedom as researchers, we chose to use the most commonly used post-labeling time window for children in our age range. However, for the sake of transparency, we also include all analyses outlined in our pre-registration in our scripts posted to OSF. First, to explore whether there were main effects of semantic connectivity, we visualized and qualitatively examined the time courses of fixation proportions to high- vs. low-connectivity items (and target vs. distractors in the test period) and conducted exploratory analyses to determine whether there were periods of significant difference using a cluster-based permutation analysis [74], implemented in R (v 3.4.2; [75]) using the eyetrackingR package (v 0.1.7; [76]). Second, to explore whether connectivity interacted with age, vocabulary percentile, or focused attention skill, we conducted (1) planned analyses of mean looking times across the time windows and (2) exploratory analyses of the latency and duration of first looks to high- vs. low-connectivity items. We conducted these analyses using linear mixed-effects modeling with subjects and items as random effects, implemented using the lme4 package (v 1.1-21; [77]). For all mixed effects models, all variables were scaled and centered, and normative degree groups were sum coded. Finally, we note here that we explored order, but determined it was not a significant factor and decided not to include it in our models to preserve power.

For both visualizations and time window analyses, we compared interest areas using the log gaze ratio measure, calculated as the log of the ratio of fixations to the relevant items: high- over low-connectivity items in the pre-labeling period and target over the distractor in the post-labeling test period. For this metric, positive values indicate a bias towards the numerator (i.e., high-connectivity item and target), negative scores indicate a bias towards the denominator (i.e., low-connectivity item and distractor), and a score of zero indicates equivalent looks between the two. This measure is commonly used to circumvent model assumption violations that result when using proportions, which are bounded between 0 and 1, e.g., [44,78,79].

### 3.2. Exploring the Influence of Semantic Connectivity on Pre-Labeling Attentional Biases

#### 3.2.1. Pre-Labeling Period Time Course and Exploratory Cluster Analyses

**Figure 4:**
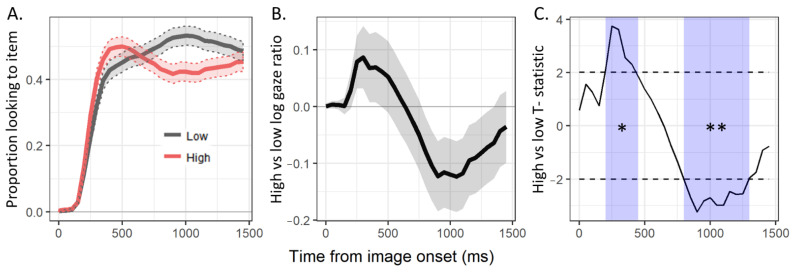
Pre-labeling period time course plots of (**A**) the proportion of fixations to the high (red) and low (grey) items, (**B**) the log gaze ratio of fixations to high vs. low items, and (**C**) the pointwise T-statistics comparing high vs. low items and periods of consecutive significant differences identified by the cluster analysis (light blue), calculated over 50 ms time bins (with *SE* ribbons). * *p* < 0.05. ** *p* < 0.01.

To determine whether there was a main effect of connectivity on pre-labeling attentional biases, we visualized and analyzed the time course of fixations to the high- and low-connectivity items in the pre-labeling period (see Figure 4).

In the time course plots, there are two apparent visual patterns. First, while fixations to both items started near zero, fixations to high-connectivity items rose more quickly than fixations to low-connectivity items. This period of early bias towards high-connectivity items was clearly seen as an initial rise in the log gaze ratio plot, and the associated period of consecutive pointwise differences (going from 200 to 450 ms post image onset) was determined to be significant according to the cluster analysis (*cluster-t* = 14.38, *cluster-p* = 0.044). Second, after the early peak, fixations towards the high-connectivity items subsided slightly and looks toward the low-connectivity items became dominant. This was also clearly represented as a negative deflection in the log gaze ratio plot, and the associated period (going from 800 to 1300 ms) was also determined to be significant (*cluster-t* = −27.02, *cluster-p* = 0.005). Together, these results reveal that connectivity did influence pre-labeling attentional biases, with a fast but brief bias towards high-connectivity items, followed by a slower but more robust bias towards low-connectivity items.

#### 3.2.2. Planned Pre-Labeling Period Time Window Analysis

Here, we determine whether the attentional biases in the pre-labeling period were related to children’s age, vocabulary percentile, or attentional focusing score. For each trial, we calculated the mean log gaze ratio of looks to high- vs. low-connectivity items over the 1500 ms pre-labeling period and entered it as the dependent variable in a linear mixed-effects model with age, vocabulary percentile, and focused attention skill as predictors and participant and item as random intercepts. This full model was then fed through a backwards stepwise feature selection algorithm (set to retain random effects) using lmerTest [80], which removes fixed effects based on *p*-values calculated at each step using the Satterthwaite approximation. No fixed effects remained in the model output from the backwards selection process, indicating there were no robust predictors of fixations to high- vs. low-connectivity items during the pre-labeling period.

#### 3.2.3. Exploratory Analyses of First Looks in Pre-Labeling Period

As an exploratory analysis, we asked whether the latency (MN = 309.7 ms, SD = 158.32 ms) and duration (MN = 673 ms, SD = 311.1 ms) of children’s first looks to items in the pre-labeling period related to item connectivity, and how connectivity interacted with children’s age, vocabulary percentile, or attentional focusing score. First, an exact binomial test revealed that children’s first looks were significantly more likely to be to the high-connectivity item (*N* = 613) than to the low-connectivity item (*N* = 538), *probability* = 0.47, *95% confidence interval* = (0.44, 0.5), *p* = 0.029. Next, we created two linear mixed-effects models with connectivity, each of the three individual differences measures, and interactions between connectivity and the individual differences measures as predictors of (1) the latency and (2) the duration of first looks. We fed both models through the stepwise feature selection algorithm, as in earlier analyses. No fixed effects remained in the model of first look latency, but the main effect of item connectivity remained in the model of first look duration (coefficients presented in Table 2). This result clarifies that the fast but brief bias towards high-connectivity items was caused by a higher probability of first looks landing on the high-connectivity item. In contrast, the slower but more robust bias towards low-connectivity items was due to the longer duration of the first looks that did land on low-connectivity items.

### 3.3. Exploring the Influence of Semantic Connectivity on Object–Label Processing

#### 3.3.1. Post-Labeling Test Period Time Course and Exploratory Cluster Analyses

**Figure 5:**
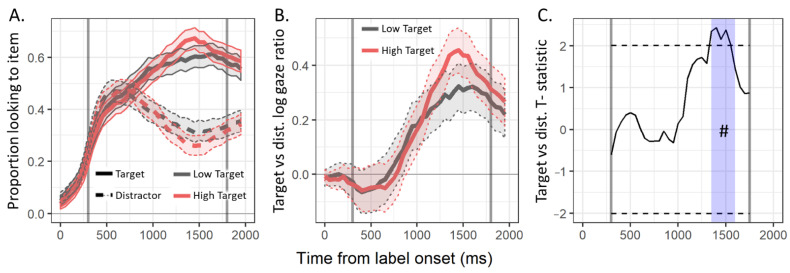
Post-labeling test period time course plots of (**A**) the proportion of fixations to the target (solid line) and distractor (dotted line) items for normative high (red) and low (grey) target conditions, (**B**) the log gaze ratio of fixations to normative high (red) vs. low (grey) items, and (**C**) the pointwise T-statistics comparing target vs. distractor log gaze ratios for high vs. low items and a period of consecutive significant differences identified by the cluster analysis (light blue), calculated over 50 ms time bins (with *SE* ribbons). # *p* < 0.1.

As with the pre-labeling period, we asked whether there was a main effect of connectivity on label processing (see Figure 5).

In the time course plots, there were once again two apparent visual patterns. First, fixations to both items started near zero, as the label was only presented once participants had fixated on the central image between the two items. Fixations to both target and distractor then began to rise quickly until around 750 ms post-label onset when fixations to distractors began to subside while fixations to the target continued to rise. This pattern is apparent in the log gaze ratio plot, with values hovering near zero until around 750 ms post-label onset, before they begin to rise, indicating a bias towards the target. Second, there was a period in the latter half of the test period in which fixations towards high-connectivity targets appeared to be greater than fixations to low-connectivity targets. The associated period of consecutive pointwise differences (going from 1350 to 1600 ms post-label onset) was determined to be marginally significant according to the cluster analysis (*cluster-t* = 11.33, *cluster-p* = 0.091). Together, these results provide tentative evidence that high connectivity facilitates label processing. 

#### 3.3.2. Post-Labeling Test Period Time Window Analysis

We next explored whether facilitated processing of high- vs. low-connectivity targets, averaged across the full test period, interacted with children’s age, vocabulary percentile, or attentional focusing score. First, similar to the pre-labeling period analysis, a linear mixed-effects model was constructed, with random intercepts for participant and item, in which the test period log gaze ratio of target vs. distractor items was predicted as a function of high vs. low connectivity, the three participant level measures (age, percentile, and focused attention skill), and the three interactions with connectivity. We also included, as a predictor, the pre-labeling period log gaze ratio of target vs. distractor items to explore the hypothesis that attentional biases during the pre-labeling period might account for connectivity effects in the test period. This full model was then fed through a backwards stepwise feature selection algorithm. Coefficients for the resulting model are presented in Table 3.

**Figure 6:**
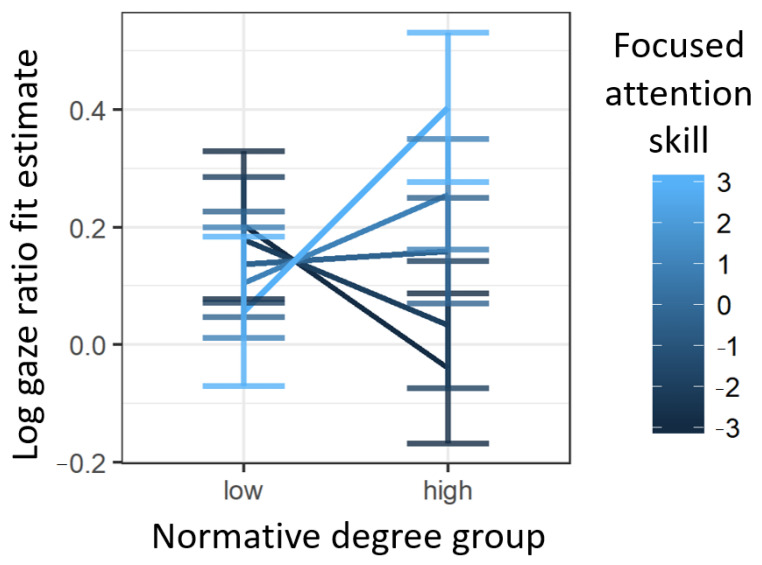
Model estimates of log gaze ratio of fixations to target vs. distractor items in the target period as a function of normative degree group and attentional focusing score.

First, the coefficient for the intercept was significantly greater than zero, quantitatively confirming that participants were recognizing and fixating on the target item. Next, the coefficient for pre-labeling looking was also significantly greater than zero, confirming that children who looked more at an item in the pre-labeling period also looked more at the same item in the post-labeling test period—as is well documented in the Intermodal Preferential Looking Paradigm literature (e.g., [81]). Finally, neither the main effects of high vs. low connectivity nor attentional focus score were significant, but their interaction was significant. This significant interaction captured the fact that children with high attentional focusing scores fixated on high-connectivity targets more than low-connectivity targets, but children with low attentional focusing scores showed the opposite pattern—as is apparent in the plot of model fit estimates of log gaze ratio of fixations to target vs. distractor items in the test period as a function of normative degree group and attentional focus scores shown in Figure 6. Crucially, this pattern was significant even when accounting for pre-labeling looking patterns.

#### 3.3.3. Exploratory Analyses of First Looks to Target in Post-Labeling Test Period

Finally, we explored whether the latency (MN = 350.31 ms, SD = 240.09 ms) and duration (MN = 1081.48 ms, SD = 521.65 ms) of first looks that landed on targets in the post-labeling test period were related to connectivity or children’s age, vocabulary percentile, or attentional focusing score. First, an exact binomial test revealed no differences in the likelihood that first looks landed on targets for high- (262) vs. low-connectivity target items (265), *probability* = 0.5, *95% confidence interval* = (0.45, 0.54), *p* = 0.93. Next, we created two linear mixed-effects models with connectivity, each of the three participant level measures, and the three interaction terms as predictors of (1) the latency and (2) the duration of first looks that landed on target items. We then fed both models through the stepwise feature selection algorithm. For the model of first look latency, no fixed effects remained. For the model of first look duration, the interaction of item connectivity with attentional focusing score and the associated main effects remained (coefficients presented in Table 4). Altogether, these results clarify how the facilitated label processing for high-connectivity items was driven in part by longer first looks to such targets, particularly for children with higher attention focusing scores.

## 4. Discussion

The current study explored how the density of a word’s perceptual connectivity affected 24 to 30-month-olds’ visual processing of objects and their subsequently spoken labels. As a secondary question, we asked whether connectivity effects varied with children’s age, word learning skill, or temperamental tendencies for focused attention. Perceptual connectivity and children’s attentional abilities emerged as significant predictors of pre- and post-labeling looking behavior.

At the outset, we hypothesized that toddlers’ higher perceptual connectivity would lead to increased attentional biases to high-connectivity objects during the pre-labeling window and would facilitate known word recognition in the post-label window. Focusing first on pre-labeling attentional biases, the results did not turn out as we predicted. Instead, we saw dynamic attentional biases that vary over the course of the pre-labeling period. Specifically, attention to high-connectivity items was fast and happened first, while attention to low-connectivity items was slow and happened second. Why did we see this result instead of the predicted outcome? The predicted outcome was largely based on empirical findings that vocabulary knowledge and category experience facilitate processing for visual objects [47,48,49]. However, while facilitation can be clearly linked to greater looking post-labeling e.g., [82], the linking hypothesis is less clear for pre-labeling looking. Recall that the studies on the development of visual object processing highlighted a shift from slower, piecemeal processing of local perceptual features to faster, more holistic processing as children gain knowledge and experience with the relevant items. Our initial predictive inference based on this progression was that in the context of two visual objects, one with high vs. one with low perceptual connectivity, higher connectivity would relate to faster processing and biased attention to the object. However, this line of thinking fails to consider the fact that the pre-labeling period is long enough (1500 ms) for children to make more than one look. Indeed, considering this fact, we might predict the opposite pattern: looking could on average be biased towards lower connectivity items due to their slower processing. In other words, there are two different pre-labeling attentional processes that could be influenced by perceptual connectivity: (1) initial likelihood of looks landing on the object and (2) the amount of time that children spend looking at the object once their eyes are there. Together, these two processes raise the possibility of dynamic attentional biases that vary over the course of the pre-labeling period—which is exactly what we saw. Exploratory analyses of first looks indicated that the initial bias towards high-connectivity items mainly resulted from a greater likelihood for first looks to land on high-connectivity items, while the later bias towards low-connectivity items was driven by longer durations for first looks that landed on low-connectivity items. Finally, regarding our secondary question, our results did not reveal any significant interactions between the effects of connectivity on pre-labeling attentional biases and any of the three individual differences.

We next investigated how perceptual connectivity influenced lexical recognition during subsequent object–label processing and its relation with pre-labeling biases. We predicted facilitated lexical recognition for high-connectivity items based on evidence that processing is facilitated for words belonging to categories that children are relatively familiar with [44], together with evidence that members of denser categories also share a greater number of shared perceptual features [7,17]. Our time course and time-window analyses both suggested that high connectivity facilitated processing. Further analyses that controlled for additional factors such as individual differences and pre-attentional biases further suggested that this connectivity effect is largely driven by participants with high Attentional Focus scores. Altogether, this pattern of results raises the possibility for both attentional and lexical pathways for perceptual connectivity in early language development.

These findings have a number of theoretical implications for early lexicosemantic development. First and foremost, our results provide the first empirical evidence that patterns of shared perceptual features influence early lexical processing—consistent with theoretical accounts highlighting the early importance of perceptual features of objects ([22,24,28,34], etc.) and work suggesting lexicosemantic connectivity often has facilitatory effects in lexicosemantic development, e.g., [44,83,84]. Second, our results highlight the potential for a newfound set of developmental dependencies linking 2-year-olds’ skill and attentional biases in visual object recognition and object name processing via the perceptual structure of their vocabularies [85]. Specifically, the parallel effects of perceptual connectivity on pre-labeling attentional biases and subsequent label processing suggest that growing perceptual connectivity in infants’ lexicosemantic networks facilitates both visual object processing—enabling faster, more holistic processing—and lexical processing. Finally, the interaction of connectivity with attentional focus score indicates that a tendency for maintaining focused attention during learning opportunities may provide crucial support for the recognition of shared perceptual features between objects. One way this could play out is via an interaction with parent speech. For example, recent work exploring how the topical content of parents’ child-directed speech in free toy play relates to their infants’ patterns of sustained attention to speech referents found the greatest proportion of sustained attention for parent utterances primarily conveying information about object features [86]. This seems to indicate that parents are shaping the content of their speech to fit their infants’ patterns of attention, and infants who are more likely to engage in focused attention may thus also be more likely to have perceptual characteristics of items highlighted by their parents. Thus, future work further exploring how individual differences in word learning outcomes relate to tendencies for sustained attention to speech targets—measured either via looking measures, e.g., [63,65], or parental questionnaire, e.g., [66,67]—might also benefit from including consideration of the perceptual connectivity of children’s vocabularies.

We next consider some limitations that constrain the scope of these findings and point the way for future directions. First, the current project only explored the role of perceptual connectivity on attentional biases and label processing in situations in which two items from the same category were presented together. Furthermore, the item categories were limited to vehicles and animals. However, if the recognition of shared perceptual features is a broadly applied mechanism in early lexicosemantic development, we would expect to find effects of perceptual connectivity in a wide variety of situations, including contexts containing sets with a greater number of items from different and wider ranging categories. Furthermore, we might expect effects to extend beyond eye fixations to include differential patterns of interaction with high- versus low-connectivity items in more ecologically valid contexts such as toy play. Second, it is difficult to determine if the results of the pre-labeling period are truly resulting from differences in connectivity or unrelated differences in relative low-level visual salience between images. While efforts were made to equate pairwise saliency of images and the use of linear mixed-effects modeling with random effects for items is designed to reduce this problem, the potential that such low-level saliency effects are obscuring perceptual connectivity effects remains. Future work exploring these effects on known-word processing might benefit from either (1) making use of visual salience mapping algorithms to include metrics of relative salience as independent variables in models, or (2) using more elaborate stimuli pairing designs—for example, using a design in which some items have high connectivity in one pair and low connectivity in another. Conversely, another way to address this issue is to use novel items so that perceptual features of objects can be systematically manipulated. Indeed, there is a growing body of work following this general approach—with examples including studies where novel items vary in either their pairwise perceptual similarity [87] or the density of their respective categories in toddlers’ productive vocabularies [83]. The results of these studies suggest toddlers are sensitive to and encode patterns of pairwise similarity in lexical representations even for newly learned words—processes that could give rise to the pattern of results revealed in the current study. Finally, building on work demonstrating that perceptual features matter most for this age range [7], this work focused on only visual–motion and visual–form and surface features. While the results support the influence of such features in early lexicosemantic processing, future work should explore a wider range of features across a wider range of ages.

## 5. Conclusions

In sum, the current project provides evidence that perceptual connectivity influences early lexicosemantic processing, including both pre-labeling attentional biases and subsequent processing of object labels. This pattern of results supports accounts emphasizing the early importance of perceptual features and provides tentative evidence that the recognition of shared perceptual features is one of the mechanisms underlying early individual differences in online lexicosemantic processing—which could, over time, relate to individual differences in productive vocabulary size and language skills more broadly.

## Figures and Tables

**Table 1:** **(A)** Descriptive statistics for age, vocabulary percentile, and focused attention skill. (**B**) Pairwise correlations for age, vocabulary percentile, and focused attention skill.

**(A)**				
**Measure**	**MN**	**SD**		**Range**
Age (months)	26.57	1.99		24–30
Vocabulary percentile	43.33	26.99		1–99
Focused attention skill	4.71	0.7		2.67–6.5
**(B)**				
**Measure**	**1**		**2**	
1. Age (months)	-		-	
2. Vocabulary percentile	−0.2		-	
3. Focused attention skill	−0.13		0.34 *	

Note: * *p* < 0.05.

**Table 2:** Results for the model output from the backwards stepwise feature selection of durations of first looks in the pre-labeling period.

Variable	Coef.	95% CI	*p* Value
Intercept	0.066	[−0.130, 0.260]	0.49
Normative High	−0.123	[−0.238, −0.008]	0.035 *

*Note*. Coef.: Coefficients are β_std_. * *p* < 0.05.

**Table 3:** Results for the model output from the backwards stepwise feature selection of log gaze ratio of fixations to target vs. distractor in the post-labeling test period.

Variable	Coef.	95% CI	*p* Value
Intercept	0.156	[0.032, 0.279]	0.034 *
Pre-labeling Log Gaze Ratio	0.08	[0.04, 0.122]	<0.001 ***
Normative High	0.052	[−0.192, 0.296]	0.69
Focused attention skill	0.025	[−0.019, 0.069]	0.27
Norm High: Focus attention skill	0.098	[0.02, 0.176]	0.013 *

*Note*. Coef.: Coefficients are β_std_. * *p* < 0.05, *** *p* < 0.001.

**Table 4:** Results for the model output from the backwards stepwise feature selection of durations of first looks in the pre-labeling period.

Variable	Coef.	95% CI	*p* Value
Intercept	−0.064	[−0.220, 0.088]	0.49
Normative High	0.133	[−0.001, 0.267]	0.051 ^#^
Attentional Focus	−0.005	[−0.124, 0.114]	0.94
Norm High: Atten Focus	0.142	[0.007, 0.277]	0.04 *

*Note*. Coef.: Coefficients are β_std_. ^#^*p* < 0.1, * *p* < 0.05.

## Data Availability

Data and analysis code are publicly available on OSF: https://osf.io/ha48g/.

## Appendix A

**Table A1:** Experimental item characteristics.

Item	Group	VM and VFS Degree	Total Degree	Percep. NoF	VM and VFS NoF	CHILDES Freq.	Number Phonemes	Number Syllables	Bigram Freq.	Phon. ND	AoA	Proportion Produces
bus	high	33	50	10	6	6.84	3	1	7.95	7	20.44	0.82
boat	low	23	31	6	1	6.1	3	1	8.69	9	20.19	0.85
truck	high	30	32	5	4	7.87	4	1	6.63	4	18.56	0.93
bike	low	10	50	9	6	5.95	3	1	7.64	8	21.19	0.81
airplane	high	55	57	8	4	6.34	5	2	5.54	0	19.74	0.90
train	low	33	36	7	4	7.31	4	1	7.13	6	20.61	0.86
bug	high	43	44	6	5	6.37	3	1	7.95	19	20.95	0.83
chicken	low	23	72	10	6	6.91	5	2	6.37	1	22.31	0.76
bear	high	44	84	11	7	7.86	3	1	8.02	18	18.59	0.87
duck	low	22	57	11	7	8.01	3	1	7.31	13	16.44	0.94
cat	high	31	34	12	9	7.38	3	1	7.33	22	17.27	0.94
fish	low	5	71	5	2	7.43	3	1	6.92	3	18.44	0.91

**Table A2:** Group-level statistics and comparisons of experimental items.

Measure	VM and VFS Degree	Total Degree	Percep. NoF	VFS and VM NoF	CHILDES Freq.	Number Phonemes	Number Syllables	Bigram Freq.	Phon. ND	AoA	Proportion Produces
High											
MN	39.33	57.33	10	6.17	1766.83	3.33	1.17	1679.07	12.33	19.01	0.89
SD	9.77	23.80	3.16	1.94	877.08	1.03	0.41	1105.31	9.89	1.11	0.05
Range	30–55	32–87	5–14	4–9	569–2621	2-5	1–2	254.93–3039.46	0–23	17.27–20-44	0.82–0.94
Low											
MN	19.33	48	7.5	4.17	1340.17	3.5	1.17	2125.07	6.67	19.64	0.87
SD	10.13	14.64	2.17	2.32	972.42	0.84	0.41	1922.36	4.32	1.85	0.05
Range	5–33	31–71	5–11	1–7	383–3005	3–5	1–2	966.16–5962.28	1–13	16.44–21.19	0.81–0.94
Comparison											
paired T-val	9.13	0.75	1.42	1.44	0.90	−0.28	0	−0.64	1.15	−0.94	0.62
*p*-value	<0.001	0.49	0.22	0.21	0.41	0.79	1	0.55	.30	.39	.56

Note: VM and VFS Degree—item degree in networks built using on visual–motion and visual–form and surface features; Percep. NoF—number of perceptual features; VFS and VM NoF—number of visual–form and surface and visual–motion features; Phon ND—Phonological Neighborhood Distance (Colheart’s N); AoA—age of acquisition; Proportion Produces—proportion of 26-month-olds that produce the word on the MBCDI:WS.
